# Efficacy of posterior fixation and bone graft fusion for treatment of lumbar brucellosis spondylitis

**DOI:** 10.1097/MD.0000000000036577

**Published:** 2023-12-15

**Authors:** Yu Li, Le Fei, Jiandang Shi

**Affiliations:** a Ningxia Medical University, Yinchuan, Ningxia, China; b Ningxia Medical University, Department of Orthopedics, General Hospital of Ningxia Medical University, Yinchuan, Ningxia, China; c Ningxia Medical University, Department of Spine Orthopedics, General Hospital of Ningxia Medical University, Yinchuan, Ningxia, China.

**Keywords:** simple fixation bone graft fusion, spinal brucellosis

## Abstract

The purpose of this study is to verify whether early stage patients with single-segment lumbar Brucella spondylitis can still be cured through simple posterior fixation and bone grafting, even without debridement. A retrospective study was conducted on 63 patients diagnosed with single-segment lumbar brucellosis spondylitis, who underwent posterior-only debridement (or not), bone grafting, and instrumentation from June 2016 to June 2019. Group A comprised 34 patients who did not undergo debridement, while group B comprised 29 patients who underwent debridement. The clinical data and imaging results of the patients were compared between the 2 groups to evaluate the clinical effects of debridement or not. Both groups of patients completed at least 1 year of follow-up. The group A had significantly lower values for operation time, blood loss, and hospital stay compared to the group B (*P* < .05). There were no significant differences between the 2 groups in terms of erythrocyte sedimentation rate, C-reactive protein, visual analogue scores, improvement of Japanese Orthopaedic Association Evaluation of treatment score, and Cobb angle. The bone fusion rate was 92% (31 patients) in group A and 96% (28 patients) in group B, with no significant difference between the 2 groups (*P* > .05). In summary, these findings suggest that posterior fixation and bone graft fusion are effective treatments for single-segment lumbar brucellosis spondylitis in early stages even without debridement. Importantly, these procedures offer several benefits, such as minimal trauma, short operation times, rapid postoperative recovery, and favorable bone graft fusion outcomes.

## 1. Introduction

Brucellosis spondylitis (BS) is an inflammatory condition caused by Brucella species that infect spinal bones, fibrocartilage discs, and ligament complexes. The disease is typically found in the lumbar spine and presents with symptoms such as fatigue, fever, and joint pains.^[1]^ However, the most prominent symptom is severe pain in the lower back.^[2]^ While oral multiple antibiotic therapy is the standard conservative treatment, several other treatment options have proven effective against BS.^[3]^ In cases where conservative treatment fails and symptoms such as persistent low back pain, cauda equina syndrome, or large abscesses develop, surgery is necessary to relieve the corresponding symptoms, especially in cases of BS that typically manifests as severe low back pain.^[4]^ Surgical methods such as posterior fixation, focus debridement, and intervertebral bone graft fusion have achieved satisfactory clinical outcomes.^[5]^ For patients with spinal infection with intervertebral abscess, debridement not only clears necrotic abscess tissue to prevent recurrence but also alleviates inflammatory reactions by clearing inflammatory factors in the intervertebral space.^[6]^ Additionally, intervertebral bone grafting can effectively restore the stability of the vertebral body in patients with vertebral destruction.^[7]^

During clinical practice, we have observed that patients with severe low back pain that is unresponsive to oral medication often exhibit only mild stenosis of the intervertebral space upon imaging examination, with some patients presenting with a small amount of purulence. For these patients, extensive debridement not only increases surgical trauma but also imposes a significant financial burden. We have found that posterior simple fixation and bone grafting, in conjunction with standard oral medication post-operation, have proven efficacious in treating severe low back pain caused by slight intervertebral space damage. As such, the clinical efficacy of posterior fixation and bone grafting in treating single-segment lumbar BS remains uncertain.

In this study, we aim to evaluate the efficacy of posterior simple fixation and bone graft fusion in the treatment of single-segment lumbar BS.

## 2. Materials and methods

### 2.1. General information

**Figure 1.**
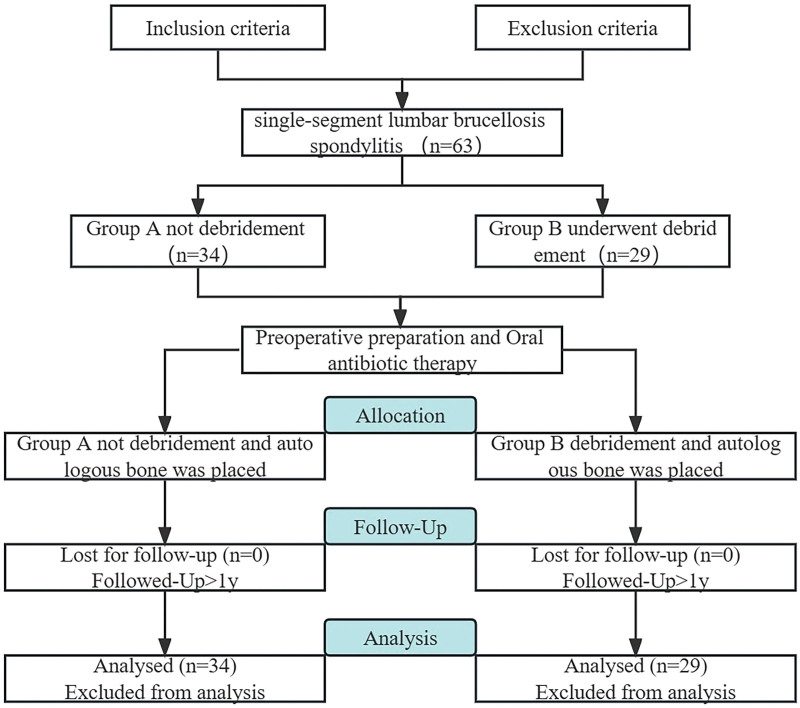
Flowchart of the trial design.

This was a retrospective study approved by the ethics committee of the General Hospital of Ningxia Medical University. All the patients have been informed of the details and requirements of the surgery and have signed the consent form. The study analyzed a total of 63 patients diagnosed with single-segment lumbar BS between June 2016 and June 2019, after applying the inclusion and exclusion criteria, we have segregated the patients into 2 groups. Group A comprised 34 patients who did not undergo debridement, while group B comprised 29 patients who underwent debridement, as shown in Figure 1.

Inclusion criteria were as follows: the lesion involved 1 adjacent lumbar segment (T1-S1); a definitive diagnosis of brucellosis was obtained through pathological examination; patients had symptoms that were not relieved after standardized conservative treatment; patients underwent debridement (or not), autologous bone grafting, and fixation through a posterior-only approach; and follow-up was completed for at least 1 year.

Exclusion criteria: multisegmental lumbar BS; other spinal diseases, such as lumbar tuberculosis or adolescent scoliosis; loss of follow-up data due to death or other reasons; and more than one-third of the vertebral body is destroyed, resulting in intervertebral instability.

### 2.2. Preoperative preparation

All patients presented with severe lower back pain, fatigue, fever, and other symptoms prior to the operation. The primary diagnosis of BS was determined based on medical history, clinical examination, laboratory results, radiologic imaging, and pathological examination. Tiger red plate and agglutination tests were conducted prior to the operation to confirm the diagnosis of BS. After completing relevant examinations and ruling out contraindications to surgery, such as heart, lung, and abdominal issues, each patient continued to receive antimicrobial therapy comprising doxycycline 0.2 g/d combined with rifampicin 0.6 g/d for 1 to 3 weeks^[8]^ in order to reduce erythrocyte sedimentation rate (ESR), C-reactive protein (CRP), and body temperature. Malnutrition and hypoproteinemia were also addressed, followed by evaluation of the segment and extent of the lesion, as well as the severity of vertebral body damage based on preoperative imaging data.

### 2.3. Surgical procedures

#### 2.3.1. Group A.

**Figure 2.**
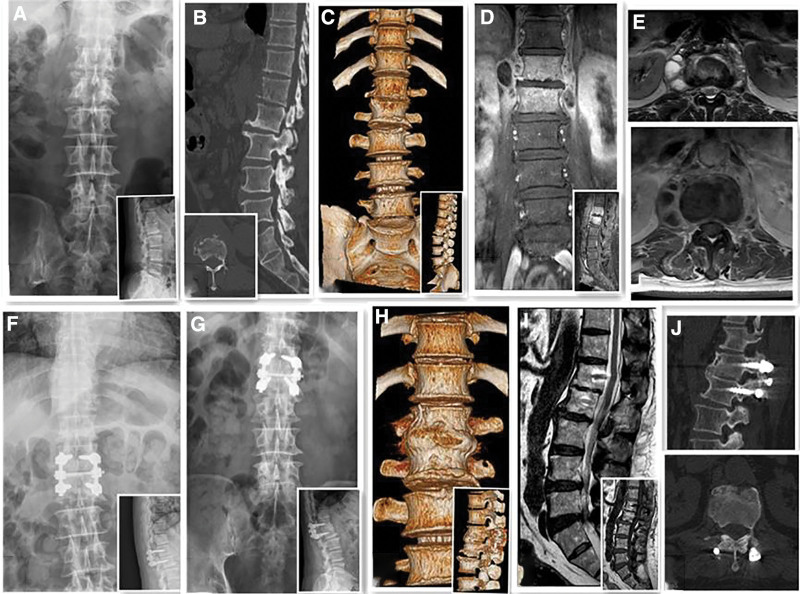
A 64-yr-old man, was admitted to the hospital mainly because of “low back pain with decreased muscle strength in both lower limbs” for 2 mo. Preoperative diagnosis revealed L1–2 brucellosis spondylitis, thus he was subjected to L1–2 simple bone graft internal fixation. (A) Preoperative X-ray films; (B and C) preoperative CT plain scan + reconstruction; (D and E) preoperative MRI; (F) X-ray films taken 3 mo after surgery; (G) lateral and frontal radiographs at last follow-up; (H and J) CT + reconstruction image at last follow-up; (I) MRI image at last follow-up.

The patients were administered general anesthesia and placed in a prone position. A sterile drape was used to dissect the skin and subcutaneous soft tissues, with the spinous process of the diseased vertebrae as the center. The electric knife was used to expose the spinous process, lamina, facet joints, and joints of the diseased segment. The pedicle needle insertion point was identified at the intersection of the midpoint of the transverse process and the outer edge of the articular process, and then opened and nailed in sequence. The pedicle needle was accurately aligned after fluoroscopy, and the connecting rod was cut and placed. Proper expansion was performed based on the degree of stenosis of the diseased segment, followed by fixation and correction. The bilateral lamina and facet joints of the diseased vertebra were decorticated to “fish scale” and autologous bone was placed. The wound was washed with normal saline, bipolar was used to stop bleeding, a drainage tube was arranged, and the wound was sutured layer by layer (Fig. 2).

#### 2.3.2. Group B.

**Figure 3.**
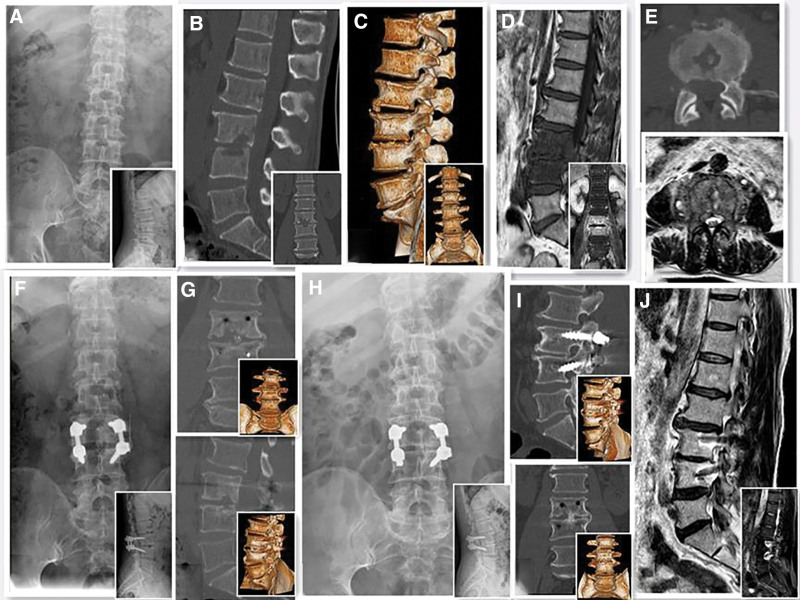
A 54-yr-old man, who was admitted to the hospital mainly because of “low back pain with activity restriction for 3 mo and worsening for 2 mo.” Preoperative diagnosis revealed L3–4 brucellosis spondylitis, thus he was subjected to posterior L3–4 lesion removal, intervertebral bone grafting, and posterior internal fixation. (A) Preoperative X-ray film; (B and C) preoperative CT plain scan + reconstruction; (D and E) preoperative MRI image; (F) X-ray film obtained at 3 mo after surgery; (G) CT plain scan + reconstruction at 3 mo after surgery; (H) postoperative final follow-up anterolateral radiograph; (I) CT plain scan + reconstruction at final follow-up; (J) MRI scan at last follow-up.

The patients were anesthetized and positioned as in group A. The diseased vertebral body was exposed layer by layer, including the spinous lamina, articular and transverse processes, using the same approach. The pedicle screw was positioned into the vertebral body as in group A, after both sides of the vertebral endplate were removed and the dural sac was gently pulled aside to locate the necrotic tissue. Pus was then removed from the dead bone until blood oozed from the bone surface of the endplate. The wound was rinsed with large amounts of normal saline. Depending on the size of the bone defect, an autologous bone from the ilium or allogeneic bone of equal size was implanted after lesion removal. The pedicle was accurately positioned via fluoroscopy, a prebent rod was installed, and the posterior screw system was properly pressurized or distracted to correct the kyphosis. A drainage tube was indwelled behind the lamina and the wound was sutured layer by layer (Fig. 3).

### 2.4. Postoperative treatment and observation

Patients were administered a combination of Doxycycline 0.2 g/d and Rifampicin 0.6 g/d antibiotics for infection prevention, starting 3 days after surgery.^[9]^ Drainage volume was monitored, and the drainage tube was removed when the volume was <50 mL. Regular antibacterial treatment was continued for 3 to 4 months post-operation. Patients were advised to wear a waist brace for 2 months when getting out of bed.

All patients underwent examinations at 3-month intervals during the first year and then every 6 months. Various parameters were recorded and compared between the 2 groups. These parameters included: Perioperative indicators such as operation times, blood loss, and hospital stay (in days); Infection indicators, including ESR and CRP; Clinical efficacy measures such as Cobb angle of kyphosis, visual analogue scores (VAS), and Japanese Orthopaedic Association Scores score; and Evaluation of bone graft fusion, which was assessed using Bridwell combined with CT bone graft fusion and instrumentation standard evaluation.^[10]^ The fusion effect of Bridwell, graded as I and II, was considered good, while the effect of grades III and IV was considered poor. Complete and incomplete fusion of CT fusion standards were considered satisfactory, while no fusion was considered poor. Image parameters, including Cobb angle and fusion status, were independently measured by 2 senior spinal surgeons. In cases of disagreement regarding graded data, the surgeons discussed the issue together.

### 2.5. Statistical analysis

Statistical analyses were conducted using SPSS 22.0 software. An independent sample t-test was used to compare the 2 groups, while non-normally distributed data were analyzed using the *X*^2^ test. One-way ANOVA was used in intragroup comparison. The Mann-Whitney test or the Chi-square test was used to analyze classification data. A *P* value of <.05 was considered statistically significant.

## 3. Result

### 3.1. Basic information

A total of 63 patients were enrolled in this study, with 34 patients in group A (no debridement) and 29 patients in group B (undergone debridement) based on the inclusion criteria. There were no significant differences in age, sex, follow-up time, lesion segment, or abscess between the 2 groups (*P* > .05). Table 1 shows the basic information of the patients in both groups.

**Table 1:** The basic information of patients in 2 groups.

Measurement	Group A(34)	Group B(29)	*P* value
Age (yr)	55.5 ± 10.3	51.2 ± 10.0	.99
Gender			.85
Male	25	23	
Female	9	6	
Follow-up (mo)	16.15 ± 2.81	16.52 ± 3.24	.63
Segments			.60
T12-L1	4	3	
L1–L2	4	1	
L2–L3	8	2	
L3–L4	6	9	
L4–L5	6	9	
L5-S1	6	5	
Abscess	12/34 (35%)	9/29 (31%)	.81

### 3.2. Comparison of perioperative indexes between groups

The operation process for patients in both groups was smooth and without complications such as vascular or nerve loss. The average operation time in group A was 102 ± 25 minutes, blood loss was 172 ± 98 mL, and hospital stay was 13.59 ± 3.61 days, which was significantly lower than that recorded in group B (*P* < .05), as shown in Table 2. All patients showed significantly lower symptoms after surgery compared to before surgery, except for 2 cases in group B and one in group A, who exhibited superficial incision swelling or partial exudation after the operation. However, these cases were healed after medication administration, and the rest of the patients recovered well.

**Table 2:** The postoperative data of patients in 2 groups.

Measurement	Group A	Group B	*P* value
Operative time (min)	102.35 ± 24.62	164.56 ± 52.67	.000
Blood loss	172.05 ± 97.84	351.72 ± 178.52	.000
Hospital stay (d)	13.59 ± 3.61	18.17 ± 5.07	.004
ESR			
Preoperative	42.21 ± 29.87	44.09 ± 28.13	.79
3 mo follow-up	9.65 ± 8.76	9.86 ± 7.05	.92
Final follow-up	5.90 ± 3.34	5.72 ± 3.53	.84
CRP			
Preoperative	23.58 ± 20.99	33.96 ± 26.21	.11
3 mo follow-up	2.73 ± 3.53	4.30 ± 6.97	.28
Final follow-up	1.25 ± 1.03	1.64 ± 1.52	.30
VAS			
Preoperative	4.50 ± 1.26	4.82 ± 1.19	.53
3 mo follow-up	1.55 ± 0.70	1.79 ± 0.77	.29
Final follow-up	1.14 ± 0.35	1.10 ± 0.31	1.66
JOA			
Preoperative	19.79 ± 2.47	18.72 ± 3.02	.13
3 mo follow-up	25.67 ± 1.36	25.31 ± 1.46	.11
Final follow-up	27.48 ± 1.15	27.93 ± 0.99	.11
Cobb (º)			
Preoperative	13.48 ± 2.28	13.63 ± 2.08	.77
3 mo follow-up	5.66 ± 0.74	5.87 ± 0.65	.21
Final follow-up	5.63 ± 0.60	5.57 ± 0.62	.41
Fusion rate	31/34 (92%)	28/29 (96%)	.13
Complications	2 (6%)	1 (3%)	.90

CRP = C-reactive protein, ESR = erythrocyte sedimentation rate, JOA = Japanese Orthopaedic Association Scores, VAS = visual analogue scores.

### 3.3. Profiles of infection indicators post-operation

Post-operative infection indexes, namely ESR and CRP, in both groups were significantly lower than those before the operation (*P* < .05). However, infection indexes for all patients returned to normal 6 months post-surgery, and the specific examination serum agglutination test titer was below 100:1. Notably, there were no statistically significant differences in infection indexes between the 2 groups before the operation, at 3 months post-operation, and at the last follow-up (*P* > .05).

### 3.4. Clinical efficacy of the operations

All 63 patients continued oral antibacterial treatment after the operation, and their liver and kidney function were monitored every 2 weeks. The drug was discontinued once the patients were cured. We found no evidence of recurrence or aggravation after the treatment. Both groups showed a return to normal VAS and Japanese Orthopaedic Association Scores 3 months after the operation and at the last follow-up, compared to before the operation (*P* < .05). The operation achieved excellent curative outcomes in both groups. There were no statistically significant differences between the groups before the operation, 3 months after the operation, and at the last follow-up (*P* > .05).

There was no evidence of internal fixation loosening or breakage in patients in both groups. Similarly, we found no adjacent spondylopathy or abnormal displacement of the autologous iliac bone during follow-up. Analysis of the Cobb angle revealed significant differences between the groups at 3 months after surgery and at the last follow-up compared to before surgery (*P* < .05). However, there was no statistically significant difference between the 2 groups at the corresponding time points (*P* > .05).

## 4. Discussion

### 4.1. Pathological characteristics of BS

The treatment of infectious spondylitis is closely related to its pathological characteristics. Brucella, classified as a type IV allergy, is the causative agent of lumbar brucella spondylitis. Pathologically, this condition is characterized by histiocytosis, proliferative nodules, and the formation of granulomas.^[11]^ Brucella moves up and down the vertebral body, and small arteries and veins spread to the vertebral body and the intervertebral disc, making lesions more common in the intervertebral disc and the upper and lower endplates of the vertebral body.^[12]^ Therefore, in comparison to tuberculous spondylitis, brucella spondylitis typically exhibits less severe bone destruction, usually limited to the area between the upper and lower vertebral endplates. The spine is a place where stress is concentrated, and prolonged wear and tear aggravate destruction of the intervertebral disc, induce instability of the diseased vertebral segment, and sustained response of local inflammation, thereby causing intractable pain in the lower back. Moreover, Brucella affects the synovial membrane and cartilage of the joints.^[13]^ This invasion is a common occurrence and has been shown to exacerbate instability of posterior facet joints, increasing the possibility of nerve roots and spinal cord compression. Pathologically, lumbar brucella spondylitis is characterized by a greater proliferation of osteoblasts compared to osteoclast destruction.^[14]^ Therefore, although the vertebral body is infected, it retains its normal status, making it easier to fixate.

Structural and morphological features are used to differentiate lumbar BS from other infectious diseases, such as tuberculosis of the spine.^[15]^ However, there are few reports of cold abscesses occurring in this condition.^[16]^ Previous studies have shown that lumbar spine BS can cause abscesses, but these are typically not accompanied by calcifications. Abscesses with a diameter larger than that of the vertebral body are rare, and there is little evidence of abscess flooding, which can cause an increase in osteoblast proliferation compared to osteoclast destruction. Additionally, the normal morphology and structure of the vertebral body makes it easy to distinguish the fusion of diseased vertebrae from tuberculous spondylitis and suppurative spondylitis, which are characterized by osteolytic destruction.

Notably, in lumbar brucella spondylitis, the vertebral body itself is less damaged and has an abundant blood supply. As a result, the formation of abscess-like lesions is rare. These factors contribute to enhanced permeability of antibacterial agents into the lesion site. In contrast, tuberculous spondylitis is characterized by the presence of a fibrous wall that restricts the diffusion of drugs at the site of the lesion. Therefore, during the early stages of development, antibacterial drugs can easily enter the lesion area, while simple fixation bone fusion can effectively treat lumbar BS. However, the infection affects the lumbar intervertebral disc and the posterior ligament complex, often causing instability of the vertebral body and aggravating lower back pain. Posterior fixation can be used to reconstruct the biomechanical stability and restore the physiological curvature of the spine.^[17]^ Reducing stimulation of soft tissues, promoting bone graft fusion, and lowering friction between facet joints can effectively relieve pain. For instance, Katonis^[18]^ found that proper internal fixation can control inflammation, promote lesion healing, and decrease the likelihood of postoperative recurrence in the surgical treatment of spinal brucellosis.

### 4.2. Insufficiency of posterior intervertebral fixation, lesion removal, and intervertebral bone graft fusion

During the removal of lesions, posterior intervertebral fixation, lesion removal, and intervertebral bone graft fusion surgery require bypassing the dural sac. This demands high technical expertise, as it can easily lead to damage to the dura and cause complications, such as cerebrospinal fluid leakage and nerve root injury.^[19]^ Furthermore, prolonged posterior landing times can worsen the occurrence of postoperative complications, such as pneumonia caused by falls and venous thrombosis in the lower extremities.^[20]^ The findings of a previous systematic review by Joaquim^[21]^ demonstrated that posterior laminectomy is an effective method for fixing the posterior column of the vertebral body, although it destroys the ligament complex between the support and the spinous process. While posterior internal fixation and intervertebral fusion can increase the stability of the diseased vertebrae, they also reduce mobility between vertebral bodies, which can exacerbate the incidence of adjacent spondylopathy. Additionally, the introduction of an anterior lesion to the rear during posterior lesion removal increases the incidence of complications, such as poor healing of the incision and sinus formation in the surgical area.

Currently, autologous iliac bone transplantation is often considered the “gold standard” for treating spinal infectious diseases. However, this procedure is time-consuming and can cause pain, iliac bone fractures, infections, and prolonged recovery time.^[22]^ For cases of single stage brucella spondylitis with minimal damage, a simple posterior bone grafting procedure can be used. This method is less invasive, has a shorter recovery time, and is almost nonirritating to the spinal cord. It can also reduce the time needed for bone grafting and fusion. Moreover, extended surgical duration and excessive blood loss during infectious spondylitis lesion clearance can impede the patient postoperative recovery and progress.^[23]^ Evidence suggests that damage to the body, low postoperative resistance, and weakened immunity increase the risk of infection recurrence.

### 4.3. Indications and limitations of posterior simple fixation and bone graft fusion

Posterior fixation and bone graft fusion may be used to treat patients with the following indications: severe lumbar pain that is not relieved by oral medication, A VAS >6; a lesion that involves a single vertebral body and causes minimal damage; a sick vertebrae with an adjacent abscess that is limited and does not exceed 5cm in diameter^[24]^; and presence of mild neurological dysfunction should be noted.

It is important to avoid performing this operation on patients with severe vertebral body damage, obvious vertebral body deformity or spondylolisthesis, and severe neurological dysfunction. In such cases, clinicians should consider removing the lesion to relieve compression while performing posterior fixation. Nerve and iliac bone can be harvested simultaneously, and intervertebral fusion can be performed to restore spinal stability.

### 4.4. Limitations

While this study yielded satisfactory outcomes, there are several limitations to consider. Firstly, it was a retrospective study, and the decision to perform debridement or not was largely based on the surgeon experience, which may have influenced the clinical outcomes of the 2 groups. Secondly, the study was conducted at a single center, with a small sample size and short-term follow-up data. Therefore, we suggest that a randomized controlled study with a larger sample size and longer follow-up period is necessary to validate the findings of this study.

## 5. Conclusion

In summary, the use of posterior simple fixation and bone graft fusion are effective treatments for single-segment lumbar BS in early stages even without debridement. Importantly, these procedures offer several benefits, such as minimal trauma, short operation times, rapid postoperative recovery, and favorable bone graft fusion outcomes.

## Acknowledgments

This is a retrospective clinical study, and the study protocol was approved by the Medical Research Ethics Review Committee of Ningxia Medical University General Hospital (KYLL-2021-676). All authors certify that the study was conducted in compliance with ethical principles. Informed consent was obtained from all participants, and written consent was obtained from each participant.

## Author contributions

**Data curation:** Yu Li, Le Fei.

**Formal analysis:** Jiandang Shi.

**Funding acquisition:** Jiandang Shi.

**Methodology:** Jiandang Shi.

**Project administration:** Yu Li, Jiandang Shi.

**Software:** Yu Li.

**Validation:** Yu Li.

**Visualization:** Yu Li.

**Writing – original draft:** Yu Li.

**Writing – review & editing:** Yu Li, Le Fei.
